# EQUIPOISE: EQUivalence In Prostate cancer treatment: surgery Or radiotherapy? An Intention-to-treat Survival analysis from real-world Evidence

**DOI:** 10.1007/s00345-026-06811-5

**Published:** 2026-10-15

**Authors:** Gustavo A. Viani, Ana Carolina Hamamura, Caio Viani Arruda, Carlos E. Cardoso, Helio A. Salmon, Gustavo O. Amaral

**Affiliations:** 1 Hospital das Clínicas of Botucatu Medical School, University of State of São Paulo (UNESP), Botucatu, SP Brazil; 2 Latin America Cooperative Oncology Group (LACOG), Porto Alegre, Brazil; 3 Master’s Program in Applied Health Sciences, University of Vassouras, Rio de Janeiro, Brazil; 4 Medical Physics, Radiocare (Oncoclínicas), Belo Horizonte, Brazil; 5 Radiotherapy Department, University of Vassouras, Rio de Janeiro, Brazil; 6 FUSVE and Business Administration Program, University of Vassouras, Rio de Janeiro, Brazil

**Keywords:** Prostatic Neoplasms, Prostatectomy, Radiotherapy, Androgen Antagonists, Propensity Score, Comparative Effectiveness Research

## Abstract

**Purpose:**

To compare cancer-specific and overall survival between a surgery-based strategy and radiotherapy plus androgen deprivation (RT + ADT) in intermediate- and high-risk localized prostate cancer, using contemporary real-world data from a Latin American public health system.

**Methods:**

Using the state-wide cancer registry of São Paulo, Brazil, we studied men with intermediate- or high-risk localized prostate cancer diagnosed in 2014–2023. Treatment was assigned by initial modality (intention-to-treat). The primary outcome was cancer-specific survival (CSS), with overall survival (OS) secondary. Groups were compared after 1:1 propensity-score matching, with weighting and per-protocol sensitivity analyses.

**Results:**

Of 21,292 patients, matching yielded 4263 pairs (all standardized mean differences below 0.10). CSS did not differ between RT + ADT and surgery [hazard ratio 0.87, 95% CI 0.58–1.29, p = 0.48 (intermediate); 1.00, 95% CI 0.78–1.27, p = 0.98 (high)]. At 5 years, CSS with surgery versus RT + ADT was 98.0% versus 96.6% (intermediate) and 94.9% versus 92.9% (high); OS was 92.6% versus 84.6% and 87.5% versus 82.7%. The crude OS advantage of surgery did not persist after matching (p = 0.10; p = 0.33). Additional therapy was given to 20.2% of operated men, rising to 48.9% in pN1 disease.

**Conclusions:**

Surgery and RT + ADT showed similar cancer-specific and overall survival after adjustment, and surgery frequently entailed multimodal therapy. Both can be presented as alternative therapeutic options in shared decision-making.

**Supplementary Information:**

The online version contains supplementary material available at https://doi.org/10.1007/s00345-026-06811-5.

## Introduction

Prostate cancer is among the most commonly diagnosed malignancies in men worldwide [1], and for clinically localized intermediate- and high-risk disease, radical prostatectomy and radiotherapy plus androgen deprivation are both guideline-endorsed curative options [2]. Whether one confers a survival advantage remains unresolved, with direct implications for counselling many men each year.

High-grade evidence is scarce, because randomized trials comparing the modalities have proved extraordinarily difficult to conduct. Several attempts, including the SPIRIT trial, closed for insufficient accrual [3], and the Scandinavian SPCG-15 trial, the first such comparison, has been feasible only within a homogeneous, tax-funded system [4, 5]. In the absence of mature randomized data, decisions rest on observational evidence, itself vulnerable to confounding by indication: patients selected for radiotherapy are typically older with more comorbidity, biasing crude comparisons in favour of surgery [6].

Two further considerations complicate interpretation. First, much observational evidence predates contemporary radiotherapy, penalizing the modality with outdated dose and technique, even though dose-escalated, image-guided radiotherapy has since improved disease control [7, 8]. Second, a surgery-based strategy is often not a single treatment, because a meaningful proportion of operated men subsequently require salvage radiotherapy and androgen deprivation, a multimodal reality that crude labels obscure [9, 10].

Evidence from Latin America is particularly limited, and to our knowledge no adjusted, population-based comparison of these modalities has been reported from the region. Using a contemporary, state-wide registry from São Paulo, Brazil, we compared cancer-specific and overall survival between a surgery-based strategy and RT + ADT in men with intermediate- and high-risk localized prostate cancer, within an intention-to-treat framework.

## Methods

### Data source and participants

**Fig. 1:**
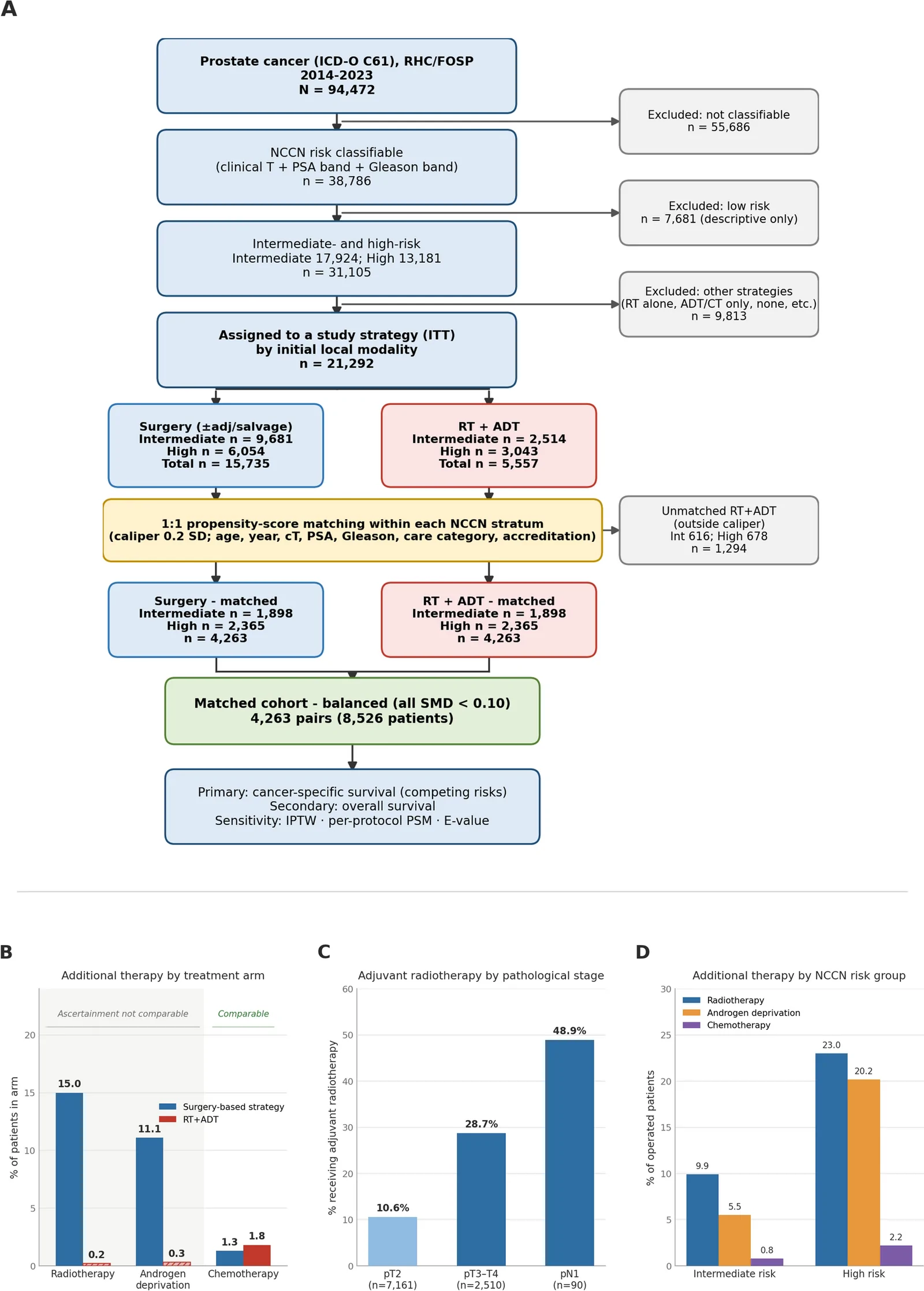
**A** Participant flow from the full registry to the intention-to-treat and propensity-score-matched cohorts. **B** Additional therapy recorded in each treatment arm; radiotherapy and androgen deprivation are not comparably ascertained between arms, whereas chemotherapy is. **C** Adjuvant radiotherapy by pathological stage within the surgical arm. **D** Additional therapy by NCCN risk group within the surgical arm

We conducted a retrospective cohort study using the Hospital-Based Cancer Registry (RHC) of the Fundação Oncocentro de São Paulo (FOSP), a state-wide registry recording demographic, tumour, staging, first-course treatment, and follow-up data (ICD-O; UICC TNM), with vital status from mortality-data linkage. Reporting follows STROBE. We included men with primary invasive prostate cancer (ICD-O C61) diagnosed in 2014–2023; the lower bound reflects systematic PSA and Gleason capture only from the TNM 7th edition (2014), making the cohort uniformly contemporary (modern dose-escalated, image-guided radiotherapy; technique and dose unrecorded). Patients were eligible if assignable to an NCCN risk group and treated primarily with surgery or radiotherapy plus androgen deprivation. The adjusted comparison was restricted to intermediate- and high-risk disease; low-risk patients were described but excluded. A participant-flow diagram details exclusions (Fig. 1A).

### Risk classification and treatment groups

Patients were stratified by a simplified three-tier, D’Amico-like scheme using clinical T category, PSA band (< 10, 10 to < 20, ≥ 20 ng/mL), and Gleason band (≤ 6, 7, ≥ 8): high risk by any of cT3-T4, PSA ≥ 20, or Gleason ≥ 8; low risk by cT1-T2a with PSA < 10 and Gleason ≤ 6; intermediate by the remainder. Treatment was assigned by initial modality (intention-to-treat). The surgery group comprised men whose primary treatment was radical prostatectomy, regardless of subsequent therapy; the RT + ADT group, men treated with external radiotherapy and androgen deprivation and no surgery, an approach shown by randomized trials to improve survival over radiotherapy alone [15]. This avoids the selection bias introduced when men requiring post-prostatectomy radiotherapy are removed from the surgical group, so the surgical arm reflects a potentially multimodal strategy. Because only the first treatment course is recorded, late salvage therapy is incompletely captured and reported proportions are lower bounds.

### Outcomes and statistical analysis

The primary outcome was cancer-specific survival (time from diagnosis to prostate-cancer death, other-cause deaths as competing events, censoring at last contact); the secondary outcome was overall survival. Survival was summarized at 3 and 5 years and truncated at 8 years with number-at-risk tables. Within each stratum, the propensity score (probability of RT + ADT versus surgery) was estimated by logistic regression including age, year, clinical T category, PSA band, Gleason band, category of care, and facility accreditation (CACON, UNACON, or other), and patients were matched 1:1 on the logit without replacement using a 0.2-SD caliper (balance assessed by standardized mean differences, < 0.10 adequate). Cancer-specific survival used the cause-specific Cox hazard, with cumulative incidence by the Aalen-Johansen method; overall survival used Cox and Kaplan–Meier. Robustness was examined with inverse-probability weighting (stabilized weights, scores truncated at the 2nd to 98th percentiles), a per-protocol matched analysis (surgery without radiotherapy), and variable-ratio matching (up to 2:1 and 3:1) with cluster-robust standard errors per matched set; for overall survival only the matched estimate was reported. Unmeasured confounding was quantified with the E-value. Hazard ratios are reported with 95% confidence intervals; two-sided p < 0.05 was significant. Analyses used Python 3.12 (lifelines, scikit-learn, SciPy, pandas).

## Results

### Cohort characteristics

Of 94,472 men registered in 2014–2023, 38,786 could be assigned an NCCN risk group; after restricting to intermediate- and high-risk disease and to surgery or RT + ADT, 21,292 formed the intention-to-treat cohort (15,735 surgery, 5,557 RT + ADT; Fig. 1A). Median follow-up was 3.4 years, with 619 prostate-cancer and 1,923 all-cause deaths.

The groups differed markedly at baseline (Table 1). RT + ADT patients were older (median 71 versus 64 years; standardized mean difference 0.92) and more often had high-risk disease (54.8% versus 38.5%), PSA ≥ 20 ng/mL (19.0% versus 8.4%), and Gleason ≥ 8 (26.3% versus 15.3%), and were more often in the public system (94.1% versus 73.9%) and at UNACON centres (43.5% versus 23.6%; surgery concentrated at CACON, 71.6% versus 54.7%). All covariates were imbalanced before matching (each p < 0.001).

**Table 1:** Baseline characteristics by treatment group, before and after propensity-score matching

Characteristic	Surgery	RT + ADT	SMD	p
Full ITT cohort (surgery n = 15,735; RT + ADT n = 5557)				
Age, median (IQR)	64 (58–69)	71 (66–75)	0.92	< 0.001
Year of dx, median (IQR)	2017 (2015–2019)	2017 (2016–2019)	0.21	< 0.001
High risk (D’Amico-like), n (%)	6054 (38.5)	3043 (54.8)	0.33	< 0.001
cT category, n (%)			0.21	< 0.001
cT1	670 (4.3)	1134 (20.4)		
cT2	10,527 (66.9)	2637 (47.5)		
cT3	4538 (28.8)	1786 (32.1)		
PSA band, n (%)			0.46	< 0.001
<10	10,987 (69.8)	2654 (47.8)		
10–20	3419 (21.7)	1848 (33.3)		
>= 20	1329 (8.4)	1055 (19.0)		
Gleason band, n (%)			0.23	< 0.001
<=6	2596 (16.5)	774 (13.9)		
7	10,732 (68.2)	3320 (59.7)		
>=8	2407 (15.3)	1463 (26.3)		
Care category, n (%)				< 0.001
SUS (public)	11,623 (73.9)	5227 (94.1)		
Private insurance	3404 (21.6)	317 (5.7)		
Out-of-pocket	708 (4.5)	13 (0.2)		
Facility accreditation, n (%)			0.35	< 0.001
CACON	11,265 (71.6)	3042 (54.7)		
UNACON	3710 (23.6)	2418 (43.5)		
Other	760 (4.8)	97 (1.7)		
Matched cohort (Surgery n = 4263; RT + ADT n = 4263)				
Age, median (IQR)	69 (65–73)	69 (65–73)	0.01	0.494
Year of dx, median (IQR)	2017 (2015–2019)	2017 (2016–2019)	0.05	0.015
High risk (D’Amico-like), n (%)	2365 (55.5)	2365 (55.5)	0.00	1.000
cT category, n (%)			0.00	0.017
cT1	488 (11.4)	549 (12.9)		
cT2	2263 (53.1)	2141 (50.2)		
cT3	1512 (35.5)	1573 (36.9)		
PSA band, n (%)			0.02	0.108
<10	2227 (52.2)	2216 (52.0)		
10–20	1291 (30.3)	1362 (31.9)		
> =20	745 (17.5)	685 (16.1)		
Gleason band, n (%)			0.01	0.065
< =6	587 (13.8)	652 (15.3)		
7	2625 (61.6)	2532 (59.4)		
> =8	1051 (24.7)	1079 (25.3)		
Care category, n (%)				<0.001
SUS (public)	4029 (94.5)	3940 (92.4)		
Private insurance	225 (5.3)	310 (7.3)		
Out-of-pocket	9 (0.2)	13 (0.3)		
Facility accreditation, n (%)			0.00	0.079
CACON	2566 (60.2)	2576 (60.4)		
UNACON	1632 (38.3)	1595 (37.4)		
Other	65 (1.5)	92 (2.2)		

Risk per simplified three-tier D’Amico-like scheme. SMD = absolute standardized mean difference (red > = 0.10; green <0.10), the primary balance metric after matching. p from Mann–Whitney U (continuous) or chi-square (categorical). Propensity score included age, year, cT, PSA, Gleason, care category and facility accreditation. CACON/UNACON = high-complexity oncology centres accredited by the Brazilian Ministry of Health. SUS = Sistema Unico de Saude, the Brazilian public (universal) health system. RT + ADT = radiotherapy plus androgen deprivation

### Treatment delivered in the surgical arm

Most surgical patients received prostatectomy alone, but 20.2% received at least one additional modality (15.0% radiotherapy, 11.1% androgen deprivation, 1.3% chemotherapy; Fig. 1B). Additional radiotherapy tracked adverse pathology, rising from 10.6% in pT2 to 28.7% in extraprostatic (pT3 to T4) and 48.9% in node-positive (pN1) disease (Fig. 1C), of whom 62.2% also received androgen deprivation. Additional therapy roughly doubled from intermediate- to high-risk disease (radiotherapy 9.9% versus 23.0%; androgen deprivation 5.5% versus 20.2%; Fig. 1D). These proportions are lower bounds.

### Unadjusted survival

Unadjusted overall survival favoured surgery in both strata (Figure S1): at 5 years, 92.6% versus 84.6% (intermediate) and 87.5% versus 82.7% (high; log-rank p < 0.001). Cancer-specific differences were much smaller (Figure S2): 98.0% versus 96.6% and 94.9% versus 92.9%, the overall-survival gap exceeding the cancer-specific gap in both strata.

### Propensity-score matched analysis

1:1 matching yielded 4263 pairs (1,898 intermediate-risk, 2365 high-risk; 1294 RT + ADT patients fell outside the caliper), achieving balance on all covariates (all standardized mean differences below 0.10; age 0.92–0.02) versus substantial pre-match imbalance (Figure S3, Table 1).

**Fig. 2:**
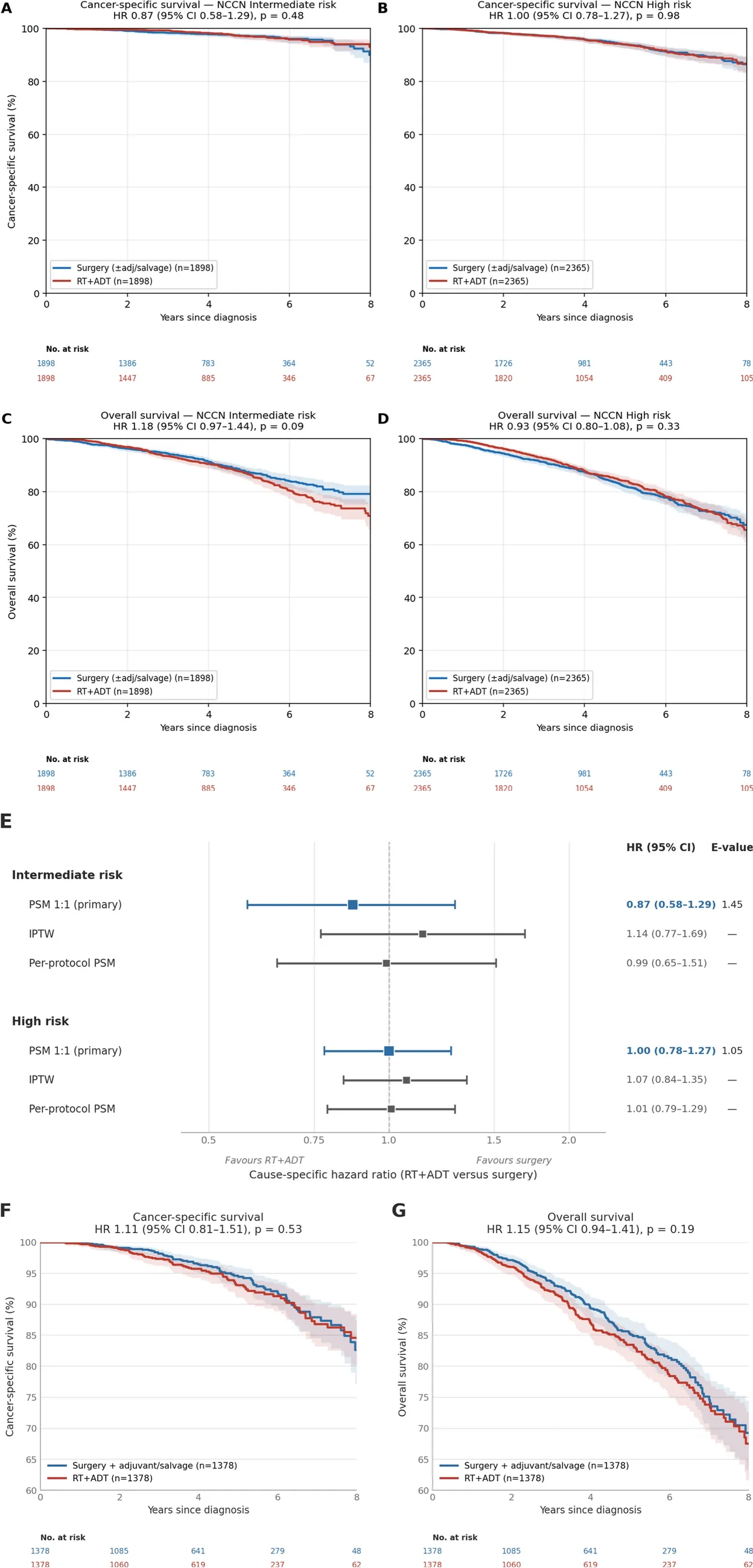
Adjusted outcomes after propensity-score matching. Cancer-specific survival (**A**, intermediate risk; **B**, high risk) and overall survival (**C**, intermediate risk; **D**, high risk), with 95% confidence intervals. **E** Cancer-specific hazard ratios across matching, weighting, and per-protocol analyses, with E-values. **F**, **G** Exploratory comparison in high-risk disease of surgery followed by adjuvant or salvage therapy versus primary RT + ADT, matched on the same propensity score

In the matched cohort, both cancer-specific and overall survival were similar between RT + ADT and surgery in each stratum, with closely overlapping curves and confidence bands (Fig. 2). The cause-specific hazard ratio for cancer death was 0.87 (95% CI 0.58–1.29; p = 0.48) in intermediate-risk disease and 1.00 (95% CI 0.78–1.27; p = 0.98) in high-risk disease (Fig. 2A, B). Overall survival likewise showed no significant difference (HR 1.18, 95% CI 0.97–1.44, p = 0.10 in intermediate-risk disease; HR 0.93, 95% CI 0.80–1.08, p = 0.33 in high-risk disease; Fig. 2C, D). Results were consistent under competing-risks modelling (Figure S4).

### Sensitivity analyses

The null cancer-specific association was consistent across approaches (Fig. 2E). With inverse-probability weighting, the cause-specific hazard ratio was 1.14 (95% CI 0.77–1.69; intermediate) and 1.07 (95% CI 0.84–1.35; high). A per-protocol analysis gave similar results (HR 0.99, 95% CI 0.65–1.51, and 1.01, 95% CI 0.79–1.29). Across approaches the hazard ratios stayed near unity (0.87–1.14), with confidence intervals always including no difference. E-values for the primary estimates were 1.45 (intermediate) and 1.05 (high). Variable-ratio matching gave concordant estimates (2:1 HR 1.04, 95% CI 0.71–1.54, and 1.07, 95% CI 0.83–1.37); confidence intervals did not narrow after within-set clustering, and balance exceeded 0.10 at 3:1 (Table S1). In an exploratory matched analysis confined to high-risk disease, RT + ADT and surgery with adjuvant or salvage therapy had similar survival (HR 1.11, 95% CI 0.81–1.51; 1378 pairs; Fig. 2F, G; Table S2).

## Discussion

In this contemporary registry cohort, surgery and RT + ADT showed similar cancer-specific survival after accounting for confounding by indication. The unadjusted advantage of surgery was confined to overall mortality, an endpoint influenced by the older age and comorbidity of patients selected for radiotherapy, whereas cancer-specific mortality did not differ. This was stable across matching, weighting, and per-protocol analysis, all yielding cause-specific hazard ratios near unity (0.87–1.14), The E-value was 1.45 for intermediate-risk but only 1.05 for high-risk disease; the high-risk null is therefore fragile, since even a weak unmeasured confounder such as comorbidity, unrecorded here, could overturn it. Crude differences thus reflected selection more than treatment.

A second finding concerns what a surgery-based strategy entailed in practice. Although most operated men received prostatectomy alone, one in five received additional therapy, rising to roughly one third in extraprostatic and nearly half in the small node-positive subgroup. Men who choose surgery should be counselled that a meaningful proportion subsequently require radiotherapy or androgen deprivation, and that surgery did not confer a cancer-specific survival advantage over upfront RT + ADT. Accurate pre-treatment staging matters: prostate-specific membrane antigen PET and multiparametric MRI can identify locally advanced or node-positive disease beforehand [16, 17], modalities largely unavailable to our cohort. Conversely, additional therapy in the RT + ADT arm was recorded in only 3.1%: salvage prostatectomy is excluded by definition and continuation of androgen deprivation is not coded as a new modality, whereas chemotherapy, ascertained identically, was similar (1.3% versus 1.8%). The contrast is therefore asymmetric by capture, not clinical. Directly comparing the multimodal surgical strategy with RT + ADT in high-risk disease showed no difference, although such groups, defined by post-operative treatment, remain selected by adverse pathology. Our endpoints are oncological only. Functional outcomes, particularly continence and potency, differ between modalities, as does the fact that a surgery-first strategy preserves salvage radiotherapy, whereas primary radiotherapy largely forecloses salvage surgery.

Our results align with the available evidence. In ProtecT, 15-year prostate-cancer death was 2.2% after prostatectomy and 2.9% after radiotherapy, in a lower-risk population treated with older radiotherapy [11]; our contemporary comparator extends this to higher-risk disease. In a propensity-matched cohort of over 20,000 men, surgery and radiotherapy had similar 5-year cancer-specific survival (97.8% versus 97.2%) but higher overall survival with surgery (96.0% versus 90.8%) [12], almost exactly our pattern, and a matched analysis in older high-risk men similarly found no cancer-specific difference [13]. A meta-analysis of 23 studies concluded that observational reports of higher radiotherapy mortality are prone to confounding and that contemporary RT + ADT approaches equivalence with prostatectomy [14]. The multimodal surgical pathway is likewise documented: adverse pathology carries up to a 60% recurrence risk at 10 years [9], and in RADICALS-RT 39% of the salvage arm received radiotherapy [10], concordant with our rates (28.7% in pT3-T4, 48.9% in pN1).

This study has limitations. Follow-up was relatively short, capturing early and intermediate rather than long-term mortality, with estimates beyond 8 years not assessable. Because prostate-cancer mortality differences classically emerge after 10–15 years, as in ProtecT, our findings indicate equivalence over the early to intermediate term, not long-term equivalence. Capturing only first-course treatment, recurrence was under-captured, precluding metastasis-free and recurrence-free endpoints. The registry lacks radiotherapy dose or technique, surgical margins, and serial PSA, and risk used a simplified D’Amico-like scheme. In particular, androgen-deprivation duration is unrecorded, so we cannot confirm that the RT + ADT arm received guideline-concordant long-course treatment, which materially affects outcomes in high-risk disease [15]. Pretreatment staging modality (CT, bone scan, MRI, or PSMA-PET) is not recorded and could not be included among matching covariates; differential understaging between arms therefore cannot be excluded. Residual confounding cannot be excluded, though consistency across methods argues against a large unmeasured effect.

The study also has strengths. To our knowledge, this is the first adjusted, population-based comparison of these strategies in a Latin American population; adjusted analyses derive from North American [12, 13], European [11], or Asian [18] datasets, with which our estimates are concordant. It provides regionally specific evidence from a publicly funded system differing in access and case-mix. Because prognostic factors were recorded only from 2014, the cohort is uniformly contemporary, countering the criticism that observational comparisons penalize radiotherapy with outdated technique. The intention-to-treat definition reflects real-world pathways and avoids the bias inflating surgical outcomes when post-prostatectomy failures are excluded.

## Conclusions

In this contemporary, population-based cohort from a Latin American public health system, surgery and RT + ADT were associated with similar cancer-specific and overall survival in intermediate- and high-risk localized prostate cancer once patient selection was accounted for. A surgery-based approach was frequently not monotherapy, as a substantial, risk-dependent proportion required adjuvant or salvage therapy. These findings support presenting both modalities as alternative therapeutic options during shared decision-making, while counselling men that surgery does not reliably avoid the subsequent need for radiation or hormonal therapy. Randomized and longer-term data remain necessary to confirm these observations.

## Supplementary Information

Below is the link to the electronic supplementary material.

Supplementary file1 (PNG 144 KB)

Supplementary file2 (PNG 140 KB)

Supplementary file3 (PNG 93 KB)

Supplementary file4 (PNG 134 KB)

Supplementary file5 (DOCX 10 KB)

Supplementary file6 (DOCX 10 KB)

## Data Availability

No datasets were generated or analysed during the current study.
