# Supplementary material for: EQUIPOISE: EQUivalence In Prostate cancer treatment: surgery Or radiotherapy? An Intention-to-treat Survival analysis from real-world Evidence
Source: World J Urol. 2026 Oct 15;44(1):749. doi: 10.1007/s00345-026-06811-5 (PMC13672427; doi:10.1007/s00345-026-06811-5)
Supplement: Supplementary file 1 [file 345_2026_6811_MOESM5_ESM.docx]

**Supplementary Table S1. Variable-ratio propensity-score matching: cancer-specific hazard ratios, balance, and precision**

*EQUIPOISE: EQUivalence In Prostate cancer treatment: surgery Or radiotherapy? An Intention-to-treat Survival analysis from real-world Evidence*

| **Matching ratio** | **NCCN stratum** | **Matched sets** | **Patients** | **Cancer deaths** | **HR (95% CI)** | **p** | **Max SMD** |
| --- | --- | --- | --- | --- | --- | --- | --- |
| **1:1 (primary)** | Intermediate | 1898 | 3796 | 96 | **0.87 (0.58–1.30)** | 0.48 | 0.057 |
| **1:1 (primary)** | High | 2365 | 4730 | 257 | **1.00 (0.78–1.28)** | 0.98 | 0.044 |
| 2:1 | Intermediate | 1615 | 4678 | 110 | 1.04 (0.71–1.54) | 0.83 | 0.099 |
| 2:1 | High | 1838 | 5302 | 258 | 1.07 (0.83–1.37) | 0.60 | 0.045 |
| 3:1 | Intermediate | 1445 | 5341 | 118 | 0.99 (0.66–1.49) | 0.97 | 0.149 |
| 3:1 | High | 1561 | 5624 | 266 | 1.07 (0.82–1.39) | 0.62 | 0.089 |

**Note.** Hazard ratios are for RT+ADT versus a surgery-based strategy (cause-specific Cox model, intention-to-treat). Controls within each matched set were weighted, and cluster-robust standard errors were applied per set. Max SMD is the largest absolute standardized mean difference across matching covariates after matching; values below 0.10 indicate adequate balance.

All ratios yielded null cancer-specific associations. Confidence intervals did not narrow with higher ratios once within-set clustering was accounted for (interval width: intermediate risk 0.72 at 1:1 versus 0.84 at 2:1; high risk 0.50 versus 0.54), because additional controls are correlated within matched sets. At 3:1, covariate balance in the intermediate-risk stratum exceeded the conventional 0.10 threshold, so 1:1 matching was retained as the primary analysis.
