# Supplementary material for: EQUIPOISE: EQUivalence In Prostate cancer treatment: surgery Or radiotherapy? An Intention-to-treat Survival analysis from real-world Evidence
Source: World J Urol. 2026 Oct 15;44(1):749. doi: 10.1007/s00345-026-06811-5 (PMC13672427; doi:10.1007/s00345-026-06811-5)
Supplement: Supplementary file 2 [file 345_2026_6811_MOESM6_ESM.docx]

**Supplementary Table S2. Exploratory comparison of a multimodal surgical strategy with RT+ADT in high-risk disease**

*EQUIPOISE: EQUivalence In Prostate cancer treatment: surgery Or radiotherapy? An Intention-to-treat Survival analysis from real-world Evidence*

Analysis confined to NCCN high-risk patients. The surgical group comprises men who received radical prostatectomy followed by adjuvant or salvage radiotherapy and/or androgen deprivation; the comparator is primary RT+ADT. Groups were matched 1:1 on the same propensity score used in the primary analysis (caliper 0.2 SD of the logit).

**Cohort**

|  | **Surgery + adjuvant/salvage** | **RT+ADT** |
| --- | --- | --- |
| Eligible, n | 1,914 | 3,043 |
| Matched, n | 1,378 | 1,378 |
| Cancer deaths, n | 76 | 81 |
| 5-year cancer-specific survival | 93.2% | 92.8% |

**Outcomes after matching (RT+ADT versus surgery + adjuvant/salvage)**

| **Endpoint** | **Hazard ratio** | **95% CI** | **p** | **Max SMD after matching** |
| --- | --- | --- | --- | --- |
| Cancer-specific survival | 1.11 | 0.81 to 1.51 | 0.53 | 0.081 |
| Overall survival | 1.15 | 0.94 to 1.41 | 0.19 | 0.081 |

**Note.** When the surgical strategy that ultimately required multimodal therapy is compared directly with primary RT+ADT, cancer-specific and overall survival are similar, consistent with the primary intention-to-treat analysis.

This comparison is exploratory. The groups are defined by receipt of post-operative treatment, a post-baseline event determined largely by adverse pathology or recurrence, so residual selection is not removed by matching. The registry does not record the timing of adjuvant or salvage therapy, precluding a formal landmark analysis. A comparison restricted to pathologically node-positive (pN1) disease was not feasible, because pathological nodal status requires lymphadenectomy and was informative in 57.7% of surgical patients versus 3.3% of RT+ADT patients (90 versus 10 patients recorded as pN1).
